# Mechanism-matched *Helicobacter pylori* treatment in the potassium-competitive acid blocker era

**DOI:** 10.3389/fmed.2026.1877827

**Published:** 2026-06-26

**Authors:** Akram Alnounou, Eric Martin Sieloff

**Affiliations:** 1 Department of Internal Medicine, Western Michigan University Homer Stryker M.D. School of Medicine, Kalamazoo, MI, United States; 2 Division of Gastroenterology, Bronson Methodist Hospital, Kalamazoo, MI, United States

**Keywords:** antimicrobial resistance, bismuth quadruple therapy, gastric cancer, *Helicobacter pylori*, molecular susceptibility testing, potassium-competitive acid blocker, rifasutenizol, vonoprazan

## Abstract

*Helicobacter pylori* infection remains common worldwide and causes chronic gastritis, peptic ulcer disease, gastric mucosa-associated lymphoid tissue lymphoma, and gastric cancer. Eradication therapy has become less reliable as resistance to clarithromycin, metronidazole, and levofloxacin has reduced the performance of older empiric regimens. Potassium-competitive acid blockers (P-CABs), molecular resistance testing, fecal susceptibility assays, and new antimicrobial strategies have changed treatment selection, but none removes the need to match regimen design to microbial biology and antibiotic activity. This narrative review argues that *H. pylori* management is shifting from empiric eradication toward mechanism-matched therapy. The framework links gastric niche biology, acid acclimation, adhesion, virulence signaling, immune evasion, resistance mechanisms, acid suppression, and partner-antibiotic selection. Recent randomized trials show the strengths and limits of this approach. Vonoprazan-based regimens improve acid suppression and can outperform proton pump inhibitor-based triple therapy in clarithromycin-resistant infection, but rescue-treatment trials show that potent acid suppression alone is insufficient when the partner antibiotic is poorly matched. Molecular testing-guided therapy and fecal molecular susceptibility testing provide practical ways to align antibiotic selection with resistance mechanisms. Rifasutenizol adds a pathogen-targeted option supported by phase 3 evidence in treatment-naive Chinese patients. Adjunctive and investigational approaches, including probiotics, vaccines, phytochemicals, phage-derived agents, and nanomedicine, should be interpreted as supportive or developmental strategies rather than replacements for validated antimicrobial regimens. A mechanism-matched approach can help clinicians and investigators choose regimens, interpret trial results, and design studies that distinguish failures caused by resistance, inadequate acid suppression, regimen intolerance, and prior antibiotic exposure.

## Introduction

*Helicobacter pylori* is one of the most common chronic bacterial infections in humans. A global systematic review and meta-analysis estimated that approximately 4.4 billion people were infected in 2015, with marked variation across regions and countries (1). A more recent meta-analysis reported that global adult prevalence declined from 52.6% before 1990 to 43.9% (95% confidence interval 42.3 to 45.5) during 2015 to 2022, although it remains high in many regions (2). The clinical burden extends beyond dyspepsia and chronic gastritis. *H. pylori* causes peptic ulcer disease and is linked to gastric mucosa-associated lymphoid tissue lymphoma and gastric adenocarcinoma (3, 4). In the GLOBOCAN 2022 estimates, stomach cancer accounted for 968,350 new cases and approximately 660,000 deaths worldwide, representing 4.9% of new cancer cases and 6.8% of cancer deaths and ranking fifth for both incidence and mortality (5). Eradication therefore serves both treatment and disease-prevention goals. However, no single regimen is uniformly optimal: available regimens differ in efficacy, tolerability, and disruption of the gastrointestinal microbiota (6, 7), and the most appropriate choice has become less stable as resistance patterns and treatment options have changed.

Older empiric regimens were built around proton pump inhibitor-based triple therapy. Their performance has declined in many settings because of resistance to clarithromycin, metronidazole, and levofloxacin, as well as prior antibiotic exposure, adherence, and intragastric acid control, all of which can affect treatment success (8, 9). The 2024 American College of Gastroenterology guideline recommends optimized bismuth quadruple therapy for treatment-naive patients when antibiotic susceptibility is unknown, with rifabutin triple therapy or P-CAB plus amoxicillin dual therapy as empiric alternatives in patients without penicillin allergy. The same guideline advises that clarithromycin- or levofloxacin-containing salvage regimens should be used only when susceptibility is confirmed (9). Maastricht VI similarly frames *H. pylori* as an infectious disease that requires resistance-aware treatment selection, susceptibility testing, and local performance data (8). These recommendations mark a clinical shift: eradication can no longer be separated from resistance diagnostics and pharmacologic context. Maastricht VI and the 2024 ACG guideline already advocate resistance-aware, susceptibility-guided selection (8, 9); the mechanism-matched framework is not intended to replace these recommendations but to make the underlying causal chain explicit and to provide a vocabulary for interpreting failure, distinguishing failures caused by an inactive partner antibiotic, inadequate acid suppression, intolerance or non-adherence, and prior exposure or regional resistance, so that trial results and individual outcomes can be read mechanistically rather than as a flat ranking of regimens.

**Figure 1:**
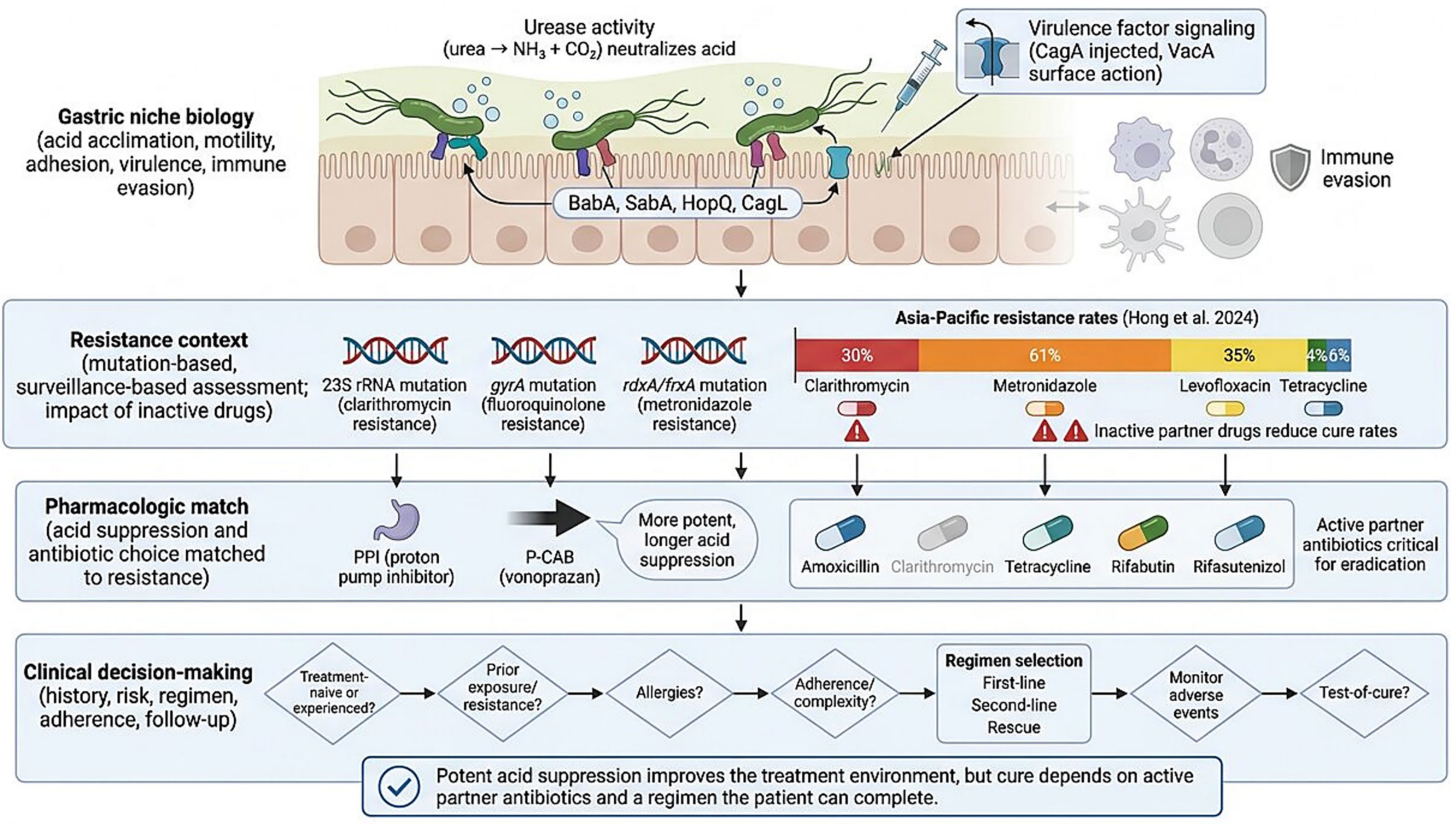
Mechanism-matched eradication model for *H. pylori*. The model links gastric niche biology, resistance context, pharmacologic matching, and clinical decision-making. *H. pylori* persistence depends on urease-mediated acid acclimation, motility through gastric mucus, epithelial adhesion, CagA and VacA-associated virulence signaling, and immune evasion. Resistance context includes mutation-associated resistance, including *23S rRNA* mutations linked to clarithromycin resistance, *gyrA* mutations linked to levofloxacin resistance, and *rdxA*/*frxA*-related mechanisms associated with metronidazole resistance, as well as regional resistance surveillance and prior antibiotic exposure. The Asia-Pacific resistance estimates shown in the figure are from Hong et al. and should be interpreted cautiously because the meta-analysis reported high heterogeneity across countries and periods. Pharmacologic matching combines adequate acid suppression with active antimicrobial components and a regimen the patient can complete. Clinical decision-making should account for treatment-naive versus rescue setting, allergy status, prior antibiotic exposure, adherence, adverse-event burden, and confirmation of eradication. BQT, bismuth quadruple therapy; P-CAB, potassium-competitive acid blocker; PPI, proton pump inhibitor.

This review synthesizes pathogenesis and therapy through a mechanism-matched framework. The main premise is that eradication regimens should reflect gastric niche biology, acid-dependent antibiotic activity, strain or population resistance, prior treatment exposure, and regimen tolerability. We use the term mechanism-matched therapy, a framing we propose here that is not yet established in the *H. pylori* literature, to describe the deliberate alignment of acid suppression, partner-antibiotic activity, strain or population resistance, prior treatment exposure, and regimen feasibility. We focus on P-CABs, molecular resistance diagnostics, recent randomized evidence, rifasutenizol, and selected adjunctive or investigational approaches. This is a narrative review, not a systematic review. It draws on consensus statements, guidelines, focused reviews, and randomized clinical trials available in the cited source set. No PRISMA screening, unpublished data, or external web sources were used. The aim is not to rank every possible regimen, but to explain how mechanism, resistance, and trial evidence should be linked when selecting and studying *H. pylori* therapy. Figure 1 summarizes the mechanism-matched framework used throughout this review.

## Gastric niche biology explains why eradication is hard

*H. pylori* persists in a compartment that is acidic, mucus-rich, and immunologically active. Long-term colonization depends on urease-mediated acid acclimation, motility through mucus, epithelial adhesion, virulence-factor delivery, and immune evasion (3, 4). These mechanisms matter therapeutically because antibiotics do not act in a neutral space. Drug stability, bacterial replication, and exposure to luminal and mucus-layer drug concentrations depend on the gastric environment.

Acid acclimation is the first treatment-relevant feature. *H. pylori* is a neutralophile that survives gastric acidity through urease-mediated generation of ammonia and carbon dioxide, buffering the periplasm and local microenvironment (3, 4). Acid suppression is therefore not only symptom control during eradication. Raising intragastric pH can improve the activity of acid-labile or growth-dependent antibiotics and increase the proportion of organisms exposed during a replicative state.

Colonization also depends on motility and adhesion. *H. pylori* uses flagella and chemotaxis to move through gastric mucus, while outer membrane adhesins, including BabA, SabA, HopQ, and CagL, support close contact with host epithelium (3, 4). These contacts sustain inflammation and allow delivery of virulence factors. The cytotoxin-associated gene pathogenicity island encodes a type IV secretion system that translocates CagA and other bacterial products into epithelial cells, promoting inflammatory signaling and epithelial disruption. VacA alters host cell function and contributes to immune escape.

Immune recognition is active but incomplete. Sirit and Peek describe altered bacterial ligands and host signaling pathways that weaken Toll-like receptor, STING, RIG-I, and NOD1 responses. They also describe changes in neutrophil chemotaxis, macrophage function, dendritic-cell phenotype, and T-cell or B-cell activity (4). These features help explain why spontaneous clearance is uncommon and why antimicrobial treatment must overcome bacterial survival programs and host immune tolerance. Therapeutically, this active but incomplete immune response means eradication cannot rely on host clearance: cure depends on delivering an active antibiotic to replicating organisms under adequate acid suppression rather than on immune assistance, so partner-antibiotic activity and acid control, not host immunity, govern success (4).

Pathogenesis does not dictate a single regimen. It explains why regimens fail in different ways. Acid suppression can improve the environment for antibiotic activity but cannot overcome resistance to a partner drug. Susceptibility testing can identify active drugs but does not solve adherence. Multi-drug regimens can broaden coverage but increase pill burden and adverse events. Mechanism-matched therapy treats these constraints as linked.

## Resistance has made empiric therapy less dependable

The decline of older triple therapy is a resistance problem interacting with pharmacology. Clarithromycin resistance is linked to *23S rRNA* mutations, and levofloxacin resistance is linked to *gyrA* mutations (8, 10). When a regimen includes an inactive antibiotic, improved acid suppression or longer treatment may not restore adequate efficacy. This is why recent guidelines distinguish empiric regimens from susceptibility-confirmed regimens (8, 9).

Hong et al. strengthen the regional-resistance argument in the Asia-Pacific region. Their updated systematic review and meta-analysis included 351 studies published between 1990 and 2022. The latest primary resistance prevalences were 30% for clarithromycin, 61% for metronidazole, 35% for levofloxacin, 4% for tetracycline, and 6% for amoxicillin, with high heterogeneity across countries and periods (11). These pooled estimates should not be applied uncritically to every clinical setting, but they show why first-line and rescue regimens must be read against local resistance and prior antibiotic exposure.

**Figure 2:**
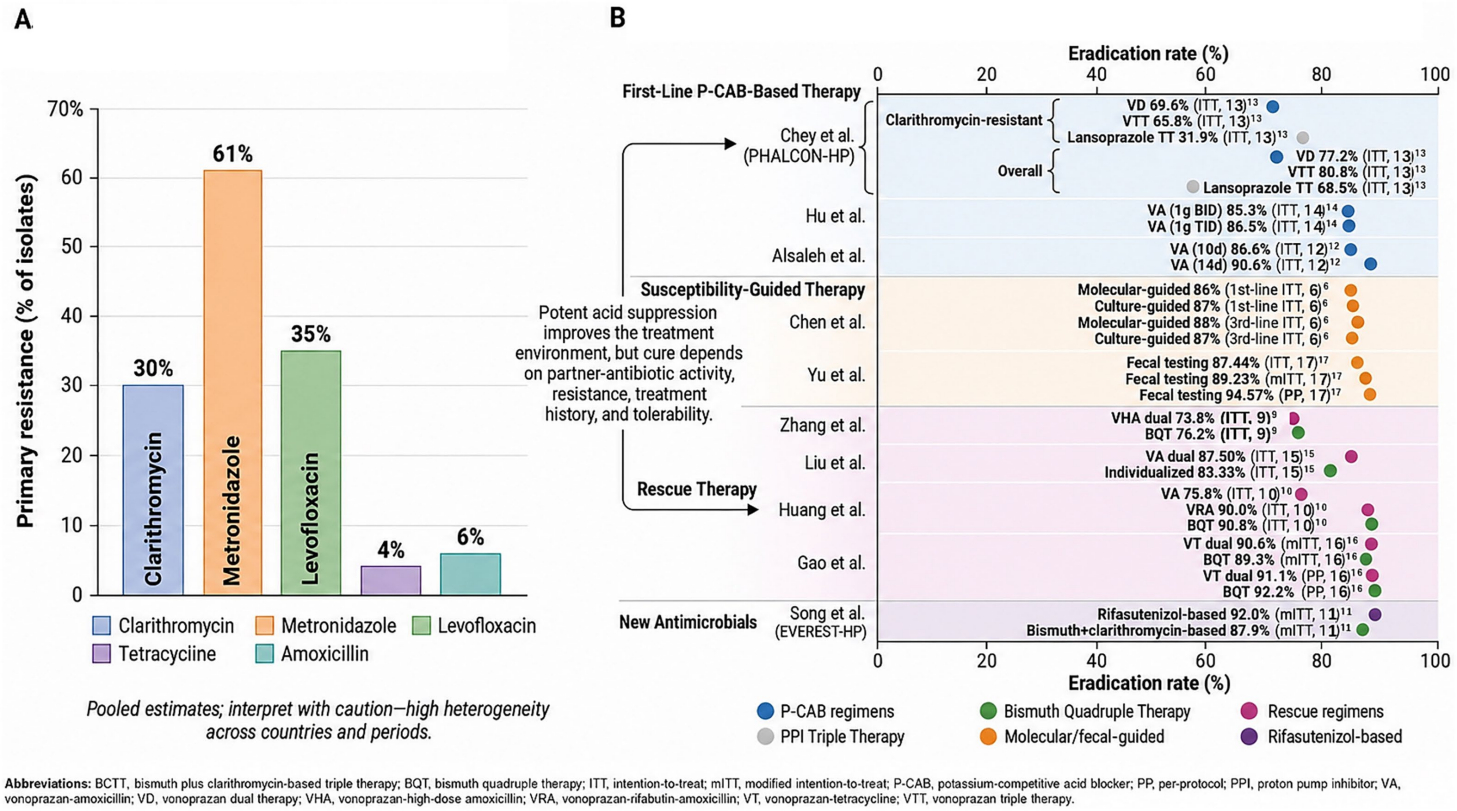
Clinical evidence snapshot for mechanism-matched *H. pylori* therapy. **(A)** Shows latest pooled Asia-Pacific primary resistance estimates from Hong et al.: clarithromycin 30%, metronidazole 61%, levofloxacin 35%, tetracycline 4%, and amoxicillin 6%; these estimates should be interpreted cautiously because heterogeneity was high across countries and periods. **(B)** Shows selected eradication rates from trials and meta-analyzes of P-CAB-based first-line therapy, molecular or fecal susceptibility-guided therapy, rescue therapy, and rifasutenizol-based therapy. Values are reported as presented in the source studies and are not pooled across trials. The Chey et al. PHALCON-HP labels use VTT for vonoprazan triple therapy and VD for vonoprazan dual therapy; the Gao et al. rescue-therapy labels use VT for vonoprazan-tetracycline dual therapy. The figure illustrates the review’s main pattern: potent acid suppression improves the treatment environment, but eradication depends on resistance context, partner-antibiotic activity, treatment history, and regimen tolerability. BCTT, bismuth plus clarithromycin-based triple therapy; BQT, bismuth quadruple therapy; ITT, intention-to-treat; mITT, modified intention-to-treat; P-CAB, potassium-competitive acid blocker; PP, per-protocol; PPI, proton pump inhibitor; RTT, rifasutenizol triple therapy; VA, vonoprazan-amoxicillin; VD, vonoprazan dual therapy; VHA, vonoprazan-high-dose amoxicillin; VRA, vonoprazan-rifabutin-amoxicillin; VT, vonoprazan-tetracycline; VTT, vonoprazan triple therapy.

Global resistance summaries point in the same direction. Yu et al. reported global primary antibiotic resistance in the recent decade, and the trial literature repeatedly shows that resistance profiles differ by region, prior treatment history, and antibiotic class (12–15). The practical message is narrow but important: empiric therapy remains necessary in many settings, but it should be empiric within a known resistance context, not empiric by habit. Resistance is also not always uniform within a host. Dual or multidrug resistance, for example combined clarithromycin and levofloxacin resistance, and heteroresistance, in which susceptible and resistant subpopulations coexist or differ between the gastric antrum and corpus, are increasingly recognized contributors to treatment failure (16–18). These phenomena reinforce the mechanism-matched argument: an empiric choice can fail not only because regional resistance is high but because a single biopsy site or a single assumption may not capture the resistance actually present, which is a further reason to favor susceptibility-aware selection when clarithromycin or levofloxacin is considered. Table 1 summarizes the key resistance and clinical evidence supporting this framework, and Figure 2 displays selected resistance estimates and trial eradication rates without pooling across studies.

**Table 1:** Clinical evidence supporting mechanism-matched *H. pylori* therapy.

Study	Setting, design, and sample size	Regimens or comparison	Main verified finding	Interpretation for this review
Chey et al. (21)	U.S./Europe phase 3 randomized trial in 1046 treatment-naive adults	Vonoprazan dual therapy, vonoprazan triple therapy, lansoprazole triple therapy	Clarithromycin-resistant infection: 69.6, 65.8, and 31.9% eradication, respectively. Overall: 77.2, 80.8, and 68.5%	P-CABs improve outcomes compared with PPI triple therapy, but cure rates still vary by resistance context
Hong et al. (11)	Asia-Pacific systematic review and meta-analysis of 351 studies	Primary resistance surveillance	Latest resistance: 30% clarithromycin, 61% metronidazole, 35% levofloxacin, 4% tetracycline, 6% amoxicillin	Empiric therapy must be interpreted locally
Chen et al. (10)	Taiwan randomized trials in first-line and third-line therapy (560 first-line; 320 third-line)	Molecular testing-guided versus culture susceptibility-guided therapy	First-line eradication 86% versus 87%; third-line 88% versus 87%	Molecular resistance testing can guide therapy, especially in refractory infection
Hu et al. (22)	China multicenter randomized first-line trial (*n* = 504; 252 per group)	Vonoprazan plus amoxicillin 1 g twice daily versus 1 g three times daily	Intention-to-treat eradication 85.3% versus 86.5%; low-dose therapy was non-inferior	Strong acid suppression may allow lower amoxicillin exposure in selected first-line settings
Yu et al. (26)	China three-arm randomized first-line trial (*n* = 598)	Empiric bismuth quadruple therapy, history-tailored therapy, fecal molecular susceptibility-tailored therapy	Fecal testing group: 87.44% ITT, 89.23% mITT, 94.57% PP	Noninvasive resistance testing can improve antibiotic matching without increasing adverse events
Liu et al. (24)	China randomized rescue trial (*n* = 240)	Vonoprazan-amoxicillin dual therapy versus drug sensitivity-based individualized therapy	Eradication was 87.50% versus 83.33%; adverse reactions were lower with dual therapy	P-CAB-amoxicillin dual therapy may be useful in selected retreatment populations
Zhang et al. (13)	China multicenter randomized rescue trial (*n* = 688)	Vonoprazan-high-dose amoxicillin dual therapy versus tetracycline-furazolidone bismuth quadruple therapy	ITT eradication was 73.8% versus 76.2%; adverse events were 13.4% versus 28.5%	Simplified rescue therapy can reduce adverse events but may not reach ideal cure rates
Huang et al. (14)	China three-center randomized rescue trial in 360 patients with at least two prior failures	10-day vonoprazan-amoxicillin, vonoprazan-rifabutin-amoxicillin, or bismuth quadruple therapy	ITT eradication was 75.8, 90.0, and 90.8%; adverse events were 8, 25, and 53%	Rescue therapy exposes the limit of acid suppression alone when partner activity is inadequate
Gao et al. (25)	China randomized rescue trial in 350 adults with at least one prior failure	14-day vonoprazan-tetracycline dual therapy versus bismuth quadruple therapy	mITT eradication was 90.6% versus 89.3%; adverse events were 10.9% versus 45.7%; adherence was 96.0% versus 87.4%	An active low-resistance partner antibiotic can support simpler therapy under potent acid suppression
Song et al. (15), EVEREST-HP	China phase 3 randomized, double-blind trial in treatment-naive adults (*n* = 700; RTT 353, BCTT 347)	Rifasutenizol-amoxicillin-rabeprazole versus bismuth-clarithromycin-amoxicillin-rabeprazole	mITT eradication 92.0% versus 87.9%; adverse events 37% versus 53%	New antimicrobial design may address some resistance constraints in selected populations

## P-CABs improve the pharmacologic environment but do not replace susceptibility

Proton pump inhibitors are metabolized principally by CYP2C19, and genotype strongly influences drug exposure: rapid and ultrarapid metabolizers achieve lower PPI exposure, which is associated with inadequate acid suppression and reduced eradication success, including for *H. pylori* (19). The frequencies of CYP2C19 alleles vary substantially by ancestry, with the increased-function *17 allele and the loss-of-function *2 and *3 alleles differing across European, Asian, and Oceanian populations (19), so PPI-based eradication data from one population may not transfer to another. P-CABs changed *H. pylori* therapy by improving the acid-suppression component of eradication regimens. Vonoprazan blocks the gastric H+/K + -ATPase through potassium-competitive inhibition and produces rapid, sustained acid suppression that, unlike that of proton pump inhibitors, is largely independent of parietal-cell activation state and CYP2C19 genotype (8, 20). This matters because amoxicillin and clarithromycin activity is influenced by intragastric pH and bacterial growth state.

The PHALCON-HP trial provides the main U.S. and European randomized anchor. In this phase 3 trial, 1,046 treatment-naive adults were randomized to 14 days of vonoprazan dual therapy, vonoprazan triple therapy, or lansoprazole triple therapy (21). In clarithromycin-resistant infection, eradication was 65.8% with vonoprazan triple therapy, 69.6% with vonoprazan dual therapy, and 31.9% with lansoprazole triple therapy. In all patients, eradication was 80.8, 77.2, and 68.5%, respectively. Treatment-emergent adverse event frequency was similar across groups (21).

The same trial also shows the limit of P-CABs. Vonoprazan improved outcomes compared with lansoprazole triple therapy, but cure rates were not uniformly high. The key interpretation is not that P-CABs solve resistance. It is that they improve the acid environment in which active antibiotics can work.

The amoxicillin-dose question supports this interpretation. Hu et al. compared vonoprazan 20 mg twice daily plus amoxicillin 1 g twice daily with vonoprazan 20 mg twice daily plus amoxicillin 1 g three times daily for 14 days in treatment-naive adults at 12 centers in China (22). Intention-to-treat eradication was 85.3 and 86.5%, respectively, and low-dose amoxicillin therapy was non-inferior to high-dose therapy. Gut microbiota diversity fell after treatment and returned to baseline by week 8 to 10 in both groups, while beta-lactam-related resistance genes returned to pretreatment levels by week 8 to 10 in the low-dose group but not the high-dose group. More antibiotic was not automatically better when acid suppression was strong.

Duration also remains context dependent. Alsaleh et al. analyzed seven open-label randomized trials from China with 3,731 treatment-naive patients (20). In vonoprazan-amoxicillin dual therapy, 10-day therapy had lower eradication than 14-day therapy in intention-to-treat analysis, 86.6% versus 90.6%, and per-protocol analysis, 89.3% versus 93.4%. In vonoprazan-based quadruple therapy, eradication did not differ by duration. Their conclusion is limited by geography because all included trials were from China. Whether this difference is clinically meaningful depends on the non-inferiority margin. A 2026 systematic review and meta-analysis that specifically tested non-inferiority found that 10-day vonoprazan-amoxicillin dual therapy was non-inferior to 14-day therapy: in the intention-to-treat population (*n* = 2,587) the pooled risk difference was −0.03 (95% confidence interval −0.06 to −0.01), with the lower confidence limit above the prespecified −0.10 margin, and the per-protocol analysis (*n* = 2,358) was concordant, with no significant differences in adverse events or compliance (23). For the mechanism-matched framework, the simplification-versus-efficacy trade-off for vonoprazan-amoxicillin duration is therefore small and may be acceptable where amoxicillin is likely active, but because the point estimate still favors 14 days, duration should be weighed against adherence and local resistance rather than treated as settled.

## Optimized bismuth quadruple therapy remains the empiric anchor

Optimized bismuth quadruple therapy (BQT) is the ACG-recommended first-line empiric regimen when antibiotic susceptibility is unknown (9). Its mechanism-matched rationale is that it does not depend on clarithromycin or levofloxacin susceptibility: it combines a proton pump inhibitor or P-CAB with bismuth, tetracycline, and metronidazole, and metronidazole resistance can frequently be overcome by adequate dose, a full 14-day course, and the additive antibacterial action of bismuth (8, 9). Optimization therefore centers on adequate dosing, 14-day duration, and adherence support. The principal limits are pill burden and tolerability and, importantly, regional availability: bismuth (subcitrate or subsalicylate), tetracycline, and fixed-dose bismuth combinations are not available in all countries, and where bismuth is unavailable the guideline alternative is non-bismuth concomitant quadruple therapy (8). These availability constraints are part of the feasibility dimension of mechanism matching rather than a detail external to it.

## Rescue trials identify partner-antibiotic selection as the rate-limiting step

Rescue therapy is the clearest test of whether acid suppression alone is enough. Patients with persistent infection after prior eradication often have higher resistance burdens and prior antibiotic exposure. Recent vonoprazan-era rescue trials show that the partner antibiotic determines whether improved acid suppression becomes reliable cure.

Zhang et al. tested vonoprazan and high-dose amoxicillin dual therapy for rescue treatment in a multicenter randomized trial in China (13). Patients who had failed previous treatment were assigned to 14-day vonoprazan-high-dose amoxicillin dual therapy or tetracycline- and furazolidone-based bismuth quadruple therapy. Eradication was 73.8% versus 76.2% by intention-to-treat analysis, 81.9% versus 85.6% by modified intention-to-treat analysis, and 82.1% versus 85.6% by per-protocol analysis. The vonoprazan-amoxicillin regimen was non-inferior and had fewer adverse events, 13.4% versus 28.5%. Multiple previous eradication failures reduced efficacy.

Liu et al. compared 14-day vonoprazan-amoxicillin dual therapy with drug sensitivity-based individualized therapy in 240 adults who had failed prior treatment (24). Eradication was 87.50% with vonoprazan-amoxicillin and 83.33% with individualized therapy in both intention-to-treat and per-protocol analyzes in the source abstract, and vonoprazan-amoxicillin met non-inferiority criteria. Adverse reactions were lower with vonoprazan-amoxicillin, and previous clarithromycin-containing treatment was an independent risk factor for clarithromycin resistance.

Huang et al. tested 10-day vonoprazan-amoxicillin dual therapy, vonoprazan-rifabutin-amoxicillin triple therapy, and bismuth quadruple therapy in 360 refractory patients with at least two previous failures (14). Intention-to-treat eradication was 75.8, 90.0, and 90.8%, respectively. Vonoprazan-amoxicillin did not meet non-inferiority to bismuth quadruple therapy, while vonoprazan-rifabutin-amoxicillin did. Resistance rates were 10% for amoxicillin, less than 1% for rifabutin, 0% for tetracycline, and 96% for metronidazole. Adverse events occurred in 8, 25, and 53% of the three groups.

Gao et al. evaluated 14-day vonoprazan-tetracycline dual therapy against bismuth quadruple therapy in 350 adults with at least one prior eradication failure (25). In modified intention-to-treat analysis, eradication was 90.6% with vonoprazan-tetracycline and 89.3% with bismuth quadruple therapy, meeting non-inferiority. Per-protocol eradication was 91.1 and 92.2%. Treatment-emergent adverse events were less frequent with vonoprazan-tetracycline, 10.9% versus 45.7%, and adherence was higher, 96.0% versus 87.4%.

These rescue trials argue against a simple hierarchy in which dual therapy is always weaker or quadruple therapy is always stronger. The relevant question is whether the partner antibiotic is active in the treated population and whether the patient can complete the regimen. A four-drug regimen containing inactive or poorly tolerated drugs may fail. A two- or three-drug regimen with potent acid suppression and active partner drugs may perform well.

## Molecular resistance diagnostics connect mechanism to treatment selection

Resistance diagnostics turn mechanism into treatment selection. Culture-based susceptibility testing remains informative, but it requires endoscopy, transport under appropriate conditions, successful culture, and laboratory capacity. Molecular testing identifies resistance-associated mutations from gastric biopsy or stool samples and can be faster and more practical in some settings (10, 26).

Chen et al. directly compared molecular testing-guided therapy with culture-based susceptibility testing-guided therapy in two multicenter randomized trials in Taiwan (10). Trial 1 enrolled 560 treatment-naive patients; trial 2 enrolled 320 patients who had failed at least two eradication therapies. In first-line treatment, eradication was 86% with molecular testing-guided therapy and 87% with susceptibility testing-guided therapy by intention-to-treat analysis. The difference was −0.7% with a 95% confidence interval of −6.4 to 5.0, and non-inferiority did not meet the prespecified 5% margin. In third-line treatment, eradication was 88% with molecular testing-guided therapy and 87% with susceptibility testing-guided therapy. The difference was 1.3% with a 95% confidence interval of −6.0 to 8.5, meeting the prespecified non-inferiority criterion.

Chen et al. also reported high detection of resistance-associated mutations among phenotypically resistant strains. In treatment-naive patients, *23S rRNA* mutation was detected in 86 of 92 clarithromycin-resistant strains and *gyrA* mutation in 77 of 92 levofloxacin-resistant strains. In refractory infection, *23S rRNA* mutation was detected in 274 of 278 clarithromycin-resistant strains and *gyrA* mutation in 197 of 210 levofloxacin-resistant strains (10). This supports molecular testing when clarithromycin or levofloxacin is being considered.

Yu et al. extended the diagnostic concept to fecal molecular susceptibility testing in first-line therapy (26). In a three-arm multicenter randomized trial, 598 treatment-naive *H. pylori*-positive patients were assigned to empirical bismuth quadruple therapy, bismuth quadruple therapy tailored by clarithromycin-use history, or bismuth quadruple therapy tailored by fecal molecular susceptibility testing. Eradication in the three groups was 82.00, 80.90, and 87.44% by intention-to-treat analysis; 82.41, 83.42, and 89.23% by modified intention-to-treat analysis; and 85.86, 87.50, and 94.57% by per-protocol analysis. The fecal susceptibility-tailored group was non-inferior to empirical therapy and was superior in the per-protocol analysis, without higher adverse events or worse compliance.

The diagnostic lesson is practical. In treatment-naive patients with reliable local data and high-performing empiric regimens, testing may not be available or necessary before every treatment. In salvage therapy, prior antibiotic exposure and higher resistance make susceptibility information more valuable. When clarithromycin or levofloxacin is being considered, molecular testing is especially relevant because the resistance mechanisms are well mapped and clinically actionable.

## Rifabutin and rifasutenizol expand the antimicrobial options

Rifabutin-based therapy avoids reliance on clarithromycin or levofloxacin susceptibility. ACG 2024 includes rifabutin triple therapy as an empiric alternative for treatment-naive patients without penicillin allergy and as an option for treatment-experienced patients in whom optimized bismuth quadruple therapy has been used previously (9). Its use still requires antimicrobial-stewardship judgment because rifabutin is also important in mycobacterial disease, including tuberculosis and nontuberculous mycobacterial infection; although rifabutin resistance in *H. pylori* is currently negligible, it is generally reserved as a rescue option to limit resistance selection and preserve activity for mycobacterial indications (9, 16).

Rifasutenizol represents a different approach: new antimicrobial design for *H. pylori*. Li et al. reported four randomized clinical trials evaluating rifasutenizol in healthy participants and Chinese patients with *H. pylori* infection (27). Rifasutenizol is a dual-targeted antibacterial agent that inhibits bacterial RNA polymerase and generates a reactive intermediate through nitroreductase action. In phase 2b regimen exploration, rifasutenizol 400 mg, rabeprazole 20 mg, and amoxicillin 1 g twice daily for 14 days achieved 95% eradication in the reported cohort. Early cohorts were small, so the findings were developmental rather than definitive.

EVEREST-HP tested rifasutenizol-based triple therapy in a phase 3 setting (15). Treatment-naive adults in China were randomized to rifasutenizol, amoxicillin, and rabeprazole or to bismuth potassium citrate, clarithromycin, amoxicillin, and rabeprazole, each for 14 days. In the modified intention-to-treat population, eradication was 92.0% in the rifasutenizol group and 87.9% in the bismuth plus clarithromycin group, with an absolute difference of 4.2% and a 95% confidence interval of −0.3 to 8.8, meeting non-inferiority. Treatment-emergent adverse events occurred in 37 and 53% of patients. Among cultured isolates, resistance was 41% for clarithromycin, 68% for metronidazole, 35% for levofloxacin, and 8% for amoxicillin, while all isolates were susceptible to rifasutenizol (15).

Rifasutenizol fits the mechanism-matched thesis because it addresses resistance through antimicrobial design rather than regimen rearrangement. The current evidence is still concentrated in China. Generalizability, comparative effectiveness, availability, cost, and stewardship need further study before broad conclusions are made.

## Adjunctive and investigational strategies should be kept in proportion

Adjunctive and non-antibiotic approaches deserve a concise section because they connect pathogenesis to future therapy. They should not be presented as substitutes for validated regimens.

Eradication regimens also perturb the host microbiota, and this is one of the trade-offs the mechanism-matched framework weighs. In the stomach, dysbiosis of the non-*H. pylori* gastric microbiota, characterized by reduced microbial diversity with enrichment of oral-type taxa, has been associated with progression of premalignant and malignant gastric lesions, so the gastric community itself may be relevant to long-term cancer risk (6). In the gut, multi-antibiotic regimens transiently reduce diversity and enrich resistance genes; in the vonoprazan-amoxicillin dual-therapy trial by Hu et al., gut diversity fell after treatment but recovered to baseline by week 8 to 10, whereas beta-lactam resistance genes returned to baseline by week 8 to 10 only in the low-dose arm (22). Probiotic co-administration with multi-antibiotic regimens has been proposed to mitigate these disturbances and treatment-related adverse effects (7). A 2025 systematic review and meta-analysis of probiotic-supplemented bismuth quadruple therapy included 19 randomized controlled trials and 2,973 samples (28). Probiotic supplementation improved eradication compared with bismuth quadruple therapy alone, with a pooled odds ratio of 1.49 and a 95% confidence interval of 1.20 to 1.85, and reduced overall adverse events, with a pooled odds ratio of 0.44 and a 95% confidence interval of 0.27 to 0.70. By contrast, ACG 2024 states that evidence is insufficient to recommend probiotics to improve eradication efficacy or tolerability in routine North American practice (9); the difference reflects strain, dose, timing, regimen, population, and certainty of evidence. Within a mechanism-matched approach, probiotics and other adjuncts are therefore best selected, like antibiotics, by matching strain, formulation, and timing to the regimen and patient, and remain supportive rather than substitutes for active antimicrobial therapy (7, 9, 28).

Vaccines remain attractive because eradication regimens cannot prevent all new acquisition or reinfection at a population level. Zeng et al. conducted a randomized, double-blind, placebo-controlled phase 3 trial of a three-dose oral recombinant *H. pylori* vaccine in children in China (29). Vaccine efficacy against infection within 1 year was 71.8%, with a 95% confidence interval of 48.2 to 85.6, among *H. pylori*-naive children in the per-protocol population. Longer follow-up was needed to confirm protection against *H. pylori*-associated disease. To date, however, no *H. pylori* vaccine has been approved for human use, and subsequent development has remained largely preclinical (30). This evidence supports prevention research, not a change in current eradication therapy.

Other strategies, including phytochemicals, phage-derived enzymes, anti-adhesion approaches, biofilm-directed agents, oxygen-niche modulation, and nanoparticle delivery, are best framed as mechanistic leads (31, 32). Current clinical treatment still rests on validated antimicrobial combinations with adequate acid suppression, tolerability, and susceptibility-aware selection. Table 2 summarizes the evidence status, strengths, and limits of current, emerging, and investigational strategies.

**Table 2:** Evidence-weighted therapeutic strategies.

Strategy	Evidence status in this review	Strengths	Main limits
Optimized bismuth quadruple therapy (8, 9)	Tier 1 (guideline-endorsed): guideline-supported empiric option when susceptibility is unknown	Broad coverage across common resistance patterns	Dosing complexity, adverse events, and drug availability
P-CAB-amoxicillin dual therapy (9, 21, 22)	Tier 1 (guideline-endorsed): randomized evidence and guideline-recognized in selected patients	Simpler regimen, avoids clarithromycin and levofloxacin	Variable rescue efficacy; depends on amoxicillin susceptibility and prior treatment
Vonoprazan triple therapy (21)	Tier 1 (guideline-endorsed): phase 3 evidence in U.S./Europe and additional trial support	Better performance than PPI triple therapy in clarithromycin-resistant subgroup	Clarithromycin-containing form still depends on susceptibility
Rifabutin-based triple therapy (9, 14, 16)	Tier 1 (guideline-endorsed): guideline-supported alternative	Avoids clarithromycin and levofloxacin	Stewardship, adverse-event, cost, and access concerns
Molecular testing-guided therapy (10)	Tier 2 (randomized-trial support, not yet guideline-standard): randomized trial evidence	Links *23S rRNA* and *gyrA* mutations to treatment choice	Platform availability, cost, and validation vary
Fecal molecular susceptibility-guided therapy (26)	Tier 2 (randomized-trial support, not yet guideline-standard): randomized first-line trial evidence	Noninvasive; improved per-protocol eradication in Yu et al. (26)	Evidence concentrated in China; implementation requires validated assays
Rifasutenizol triple therapy (15, 27)	Tier 2 (randomized-trial support, not yet guideline-standard): phase 2 and phase 3 evidence in China	New dual-targeted antimicrobial; high eradication in EVEREST-HP	Generalizability, availability, and stewardship require further study
Probiotics as adjuncts (7, 9, 28)	Tier 2 (randomized-trial support, not yet guideline-standard): meta-analysis suggests benefit; ACG remains cautious	May reduce adverse events and improve eradication in selected bismuth quadruple contexts	Strain, dose, timing, and population heterogeneity
Vaccines (29, 30)	Tier 3 (investigational): phase 3 prevention trial in children in China	Potential prevention strategy	Not current eradication therapy; disease-protection durability unresolved
Phytochemicals, phage-derived agents, nanomedicine, oxygen-niche strategies (31, 32)	Tier 3 (investigational)	Mechanistically linked to adhesion, urease, biofilm, oxygen tolerance, or delivery	Insufficient clinical evidence for routine use

## Clinical framework for mechanism-matched therapy

Mechanism-matched therapy can be organized around four questions.

First, is the patient treatment-naive or treatment-experienced? Treatment-naive patients may be eligible for empiric regimens when susceptibility is unknown, but treatment-experienced patients require careful review of prior antibiotic exposure. Prior macrolide or fluoroquinolone exposure should lower confidence in clarithromycin- or levofloxacin-containing regimens unless susceptibility is confirmed (9, 24).

Second, what is the resistance risk? If local resistance is high or unknown, optimized bismuth quadruple therapy, rifabutin-based therapy, or P-CAB-amoxicillin dual therapy may be reasonable depending on allergy status, availability, prior therapy, and guideline context (9). If clarithromycin or levofloxacin is being considered, molecular or culture-based testing should be used when available.

Third, what does acid suppression contribute? P-CABs strengthen the acid-suppression component and can improve eradication in selected settings, but they are not substitutes for active antibiotics. PHALCON-HP, Hu et al., and the 2025–2026 rescue trials all support this point from different angles (13, 14, 21, 22, 25).

**Figure 3:**
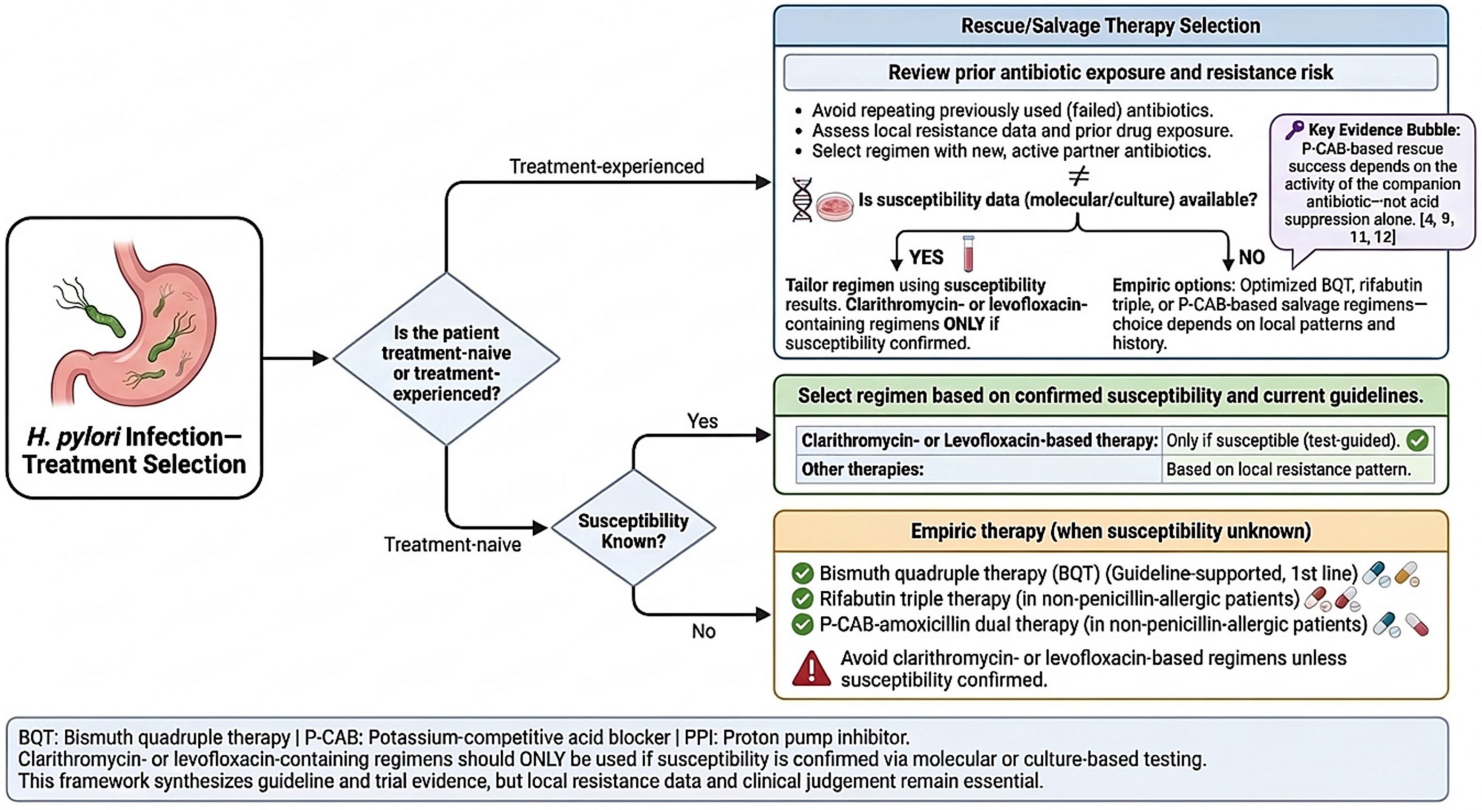
Clinical decision-making framework for mechanism-matched *H. pylori* therapy. This review-derived framework separates treatment-naive from treatment-experienced patients and integrates prior antibiotic exposure, resistance risk, susceptibility testing, allergy status, and regimen feasibility. When susceptibility is unknown in treatment-naive patients, optimized bismuth quadruple therapy is guideline-supported first-line therapy, while rifabutin triple therapy and P-CAB-amoxicillin dual therapy are empiric alternatives in patients without penicillin allergy. Clarithromycin- or levofloxacin-containing regimens should be used only when susceptibility is confirmed. In treatment-experienced patients, prior failed antibiotic classes, local resistance data, and molecular or culture-based susceptibility results should guide selection. The figure’s reference to P-CAB-based salvage therapy should be interpreted as locally supported or trial-supported rescue use rather than a universal guideline recommendation. Recent rescue trials support the framework by showing that P-CAB-based rescue regimens depend on the activity of the companion antibiotic rather than acid suppression alone. This figure is a synthesis for review purposes and does not replace local guidelines, local resistance data, or clinician judgment. BQT, bismuth quadruple therapy; P-CAB, potassium-competitive acid blocker; PPI, proton pump inhibitor.

Fourth, can the patient complete the regimen? Bismuth quadruple therapy is guideline-supported but can be complex. Simplified regimens may improve tolerability, but they should not be used when their partner antibiotic is poorly matched to the resistance risk. Mechanism matching is not regimen simplification. It is the alignment of acid suppression, antibiotic activity, treatment history, and patient feasibility. Adjunctive measures follow the same logic: where microbiota disturbance or adverse events are a concern, probiotic support can be matched to the regimen and patient rather than added reflexively, while recognizing that current guidelines do not yet endorse routine use (7, 9). Figure 3 translates this evidence into a practical decision-making framework.

## Discussion

This review argues that *H. pylori* treatment has moved into a mechanism-matched phase. The central problem is no longer only that older regimens fail more often. Empiric selection can no longer assume that acid suppression, clarithromycin, metronidazole, levofloxacin, amoxicillin, tetracycline, rifabutin, or newer agents will function the same way across populations. Gastric niche biology, resistance mechanisms, treatment history, and tolerability determine whether a regimen is plausible and effective.

P-CABs are pharmacologic enablers, not universal solutions. Chey et al. showed that vonoprazan regimens improved outcomes compared with lansoprazole triple therapy in clarithromycin-resistant infection and in the overall U.S. and European trial population (21). Hu et al. showed that lower-dose amoxicillin with vonoprazan was non-inferior to higher-dose amoxicillin as first-line therapy in China and had a less persistent beta-lactam resistome signal (22). Alsaleh et al. showed that 14-day therapy most clearly benefits vonoprazan-amoxicillin dual therapy, while their evidence came entirely from China (20). These findings support P-CAB use, but they also show that dose, duration, population, and partner antibiotic remain important.

The strongest evidence for the mechanism-matched argument comes from rescue therapy. Zhang et al., Huang et al., and Gao et al. show that simplified P-CAB-based rescue therapy can succeed or fail depending on the partner drug and resistance context (13, 14, 25). Vonoprazan-amoxicillin rescue therapy performed less well in the Huang trial, where amoxicillin resistance was 10%, while vonoprazan-rifabutin-amoxicillin and vonoprazan-tetracycline regimens performed well when the partner drug retained activity (14, 25). The lesson is not that one regimen should replace all others. It is that rescue regimens should be interpreted through active drug exposure and resistance rather than drug count alone.

Diagnostics are the operational link between mechanism and therapy. Chen et al. showed that molecular testing-guided therapy approximated culture-guided therapy in randomized trials, especially in third-line treatment (10). Yu et al. showed that fecal molecular susceptibility-tailored bismuth quadruple therapy can improve per-protocol eradication without increasing adverse events or reducing compliance (26). Hong et al. showed that Asia-Pacific primary resistance is high for clarithromycin, metronidazole, and levofloxacin but lower for tetracycline and amoxicillin, although with high heterogeneity (11). Together, these sources support broader resistance surveillance and more practical molecular testing.

New antimicrobials should be evaluated by the resistance problem they solve. Rifasutenizol is important because it was designed around a dual antibacterial mechanism and all EVEREST-HP clinical isolates were susceptible to it despite substantial resistance to several standard antibiotics (15). Its phase 3 performance in treatment-naive Chinese patients is promising, but comparative studies outside China are needed before it can be placed confidently in global algorithms.

This review has limitations. It is narrative and does not claim exhaustive screening. The evidence base is also geographically concentrated. With the exception of PHALCON-HP, conducted in the United States and Europe (21), most of the P-CAB dual and triple, rescue, diagnostic, and rifasutenizol trials discussed here were conducted in China or, for Chen et al., in Taiwan (10, 13–15, 22, 24–26). Several factors limit direct transfer of these results to North American, European, Middle Eastern, and African settings: *H. pylori* strain diversity and virulence-genotype distributions differ by region; host CYP2C19 metabolizer frequencies vary by ancestry and affect PPI-based regimens (19); diet and the gastric and gut microbiota differ (6); and resistance ecology, including primary, dual, and heteroresistance, varies widely between and within countries (11, 16–18). Hong et al. strengthen the regional-resistance argument, but their high between-study heterogeneity argues against applying pooled Asia-Pacific estimates to individual countries without local data (11). Some trials were open-label, used non-inferiority designs, and compared regimens that may not match practice in every region. These limitations are not fatal to the main conclusion because the mechanism-matched argument is supported across guidelines, pathogenesis reviews, resistance surveillance, molecular testing trials, P-CAB trials, rescue trials, and rifasutenizol development.

Future studies should answer practical questions. Which patients need susceptibility testing before first-line therapy? Which stool-based molecular platforms are accurate enough for routine use? How should P-CAB-amoxicillin dual therapy be selected by region, prior exposure, and amoxicillin resistance? When should rifabutin or rifasutenizol be preferred over optimized bismuth quadruple therapy? Which probiotic strains, if any, have reproducible benefit with modern regimens? These questions should be tested in randomized trials and implementation studies that report prior antibiotic exposure, resistance testing, dosing, adherence, adverse events, and test-of-cure.

## Conclusion

*H. pylori* eradication is moving from uniform empiric prescribing toward resistance-aware, mechanism-matched treatment. Pathogenesis explains why the organism persists in the gastric niche; resistance diagnostics explain why older empiric regimens fail; and potent acid suppression with P-CABs improves the treatment environment without removing the need for an active partner antibiotic. Operationally, the framework asks four linked questions, whether the patient is treatment-naive or treatment-experienced, what the resistance risk is, what acid suppression can and cannot contribute, and whether the patient can complete the regimen, and it treats microbiota impact and adjunctive support as part of feasibility rather than as afterthoughts. Its main value is diagnostic: it separates failures caused by an inactive partner antibiotic, inadequate acid suppression, intolerance or non-adherence, prior exposure, dual or heteroresistance, and population-specific resistance, so that clinicians and trialists can interpret why a regimen succeeded or failed rather than only which regimen to choose. Because much of the supporting trial evidence is geographically concentrated, the framework should be applied with attention to local resistance, drug availability, host pharmacogenomics, and microbiota, and validated prospectively outside East Asia. Used this way, mechanism matching offers a practical and defensible alternative to treating all eradication failures as the same event.
